# Radiomics-Based Fundus Photography Analysis in Diabetic Retinopathy

**DOI:** 10.1167/tvst.15.5.27

**Published:** 2026-05-28

**Authors:** Elham Sadeghi, Ryan Chace Williamson, Francesca Corona, Elli Davis, Michael Kozlov, Kiran Kumar Vupparaboina, Sandeep Chandra Bollepalli, José-Alain Sahel, Jay Chhablani, Evan L. Waxman

**Affiliations:** 1 Department of Ophthalmology, University of Pittsburgh, Medical Center, Pittsburgh, PA, USA; 2 Temple Medical University, Philadelphia, PA, USA; 3 SUNY Downstate Health Sciences University, Brooklyn, NY, USA

**Keywords:** radiomics, diabetic retinopathy (RD), fundus photography

## Abstract

**Purpose:**

The purpose of this study was to apply radiomics feature extraction from fundus photographs to automatically identify biomarkers that distinguish different stages of diabetic retinopathy (DR).

**Methods:**

A total of 52 radiomic features were extracted from fundus photographs representing different DR stages: diabetes without DR (*n* = 35), mild non-proliferative DR (NPDR; *n* = 33), moderate NPDR (*n* = 35), severe NPDR (*n* = 35), and proliferative DR (PDR; *n* = 34). Additionally, 68 images from 34 eyes with NPDR over 1 year of follow-up were analyzed. Statistical significance was evaluated using 95% bootstrap confidence intervals. Wilcoxon rank-sum tests, linear regression, and multi-class logistic regression were performed using R software (version 4.5.1) and Python software.

**Results:**

When comparing eyes without DR to all NPDR eyes, significant differences were detected in 6 of 16 first-order statistics (FOS), 1 of 14 gray-level co-occurrence matrix (GLCM), 13 of 16 Gabor texture (GT), and 1 of 6 Law's texture energy (LTE) features (*P* < 0.01). Several features demonstrated a linear trend with increasing DR severity, whereas PDR showed a distinct pattern. Across all 5 stages, 14 FOS, 7 GLCM, 11 GT, and 1 LTE features differed significantly (*P* < 0.05). Overall, 34 of 52 features significantly distinguished no DR from NPDR, and 17 discriminated across all stages. Longitudinally, 34 NPDR eyes showed no significant 1-year changes.

**Conclusions:**

Radiomic features from fundus photographs may help in distinguishing eyes without DR from NPDR, demonstrating strong potential for automated DR classification and screening applications.

**Translational Relevance:**

Radiomic-derived biomarkers from fundus photographs may provide automated, objective support for DR screening and staging.

## Introduction

Diabetic retinopathy (DR) is a common ocular disease in adults that can lead to blindness. In addition to retinal neurodegeneration, chronic hyperglycemia weakens the vessel walls, resulting in microvascular abnormalities such as microaneurysms, vascular leakage, exudates, and capillary occlusion, leading to fibrous tissue formation and neovascular proliferation.^1^^,^^2^ In 2020, the global prevalence of DR among individuals with diabetes was estimated at 19.7% to 25.0%, affecting approximately 103.1 million adults worldwide, which is expected to reach nearly 700 million by 2045.^3^

Early screening for DR is critical for preventing severe complications and minimizing the risk of progression to proliferative DR (PDR) or diabetic macular edema. Adoption of advanced screening technologies and timely detection can significantly reduce the global burden of DR, improve clinical outcomes, and avert vision loss in millions of individuals worldwide.^4^^,^^5^ Deep learning has markedly advanced the screening and diagnosis of DR. Trained on large datasets of retinal images, these neural networks can automatically identify disease-specific patterns and detect abnormalities across the spectrum of DR severity. These algorithms can process vast amounts of data rapidly and continuously improve as they are exposed to more data, thereby reducing the risk of human error and enhancing the efficiency of screening programs.^6^^,^^7^ In underserved and remote regions with limited access to specialists, these technologies can be integrated into telemedicine platforms, enabling non-specialist healthcare workers to conduct effective initial screenings for DR.^4^ The US Food and Drug Administration (FDA)-approved machine learning algorithms in DR are IDx-DR (Digital Diagnostics, formerly IDx Technologies) and EyeArt (Eyenuk, Inc.), which are for detecting more than mild and referable DR.^8^^,^^9^

Radiomic-based texture analysis uses standard image processing techniques to extract quantitative texture features from medical images and is widely applied in radiology and pathology.^10^^,^^11^ In recent years, its use in ophthalmology has expanded across various imaging modalities and diseases, including optical coherence tomography (OCT) and OCT angiography (OCTA), fundus photography, and fluorescein angiography, to predict, classify, and monitor the progression of DR, central serous chorioretinopathy (CSCR), age-related macular degeneration (AMD), epiretinal membrane, myopic maculopathy, and optic neuropathies.^12^^–^^19^ However, most of these efforts have concentrated on retinal layer analysis in OCT scans, with comparatively limited attention given to fundus photographs, despite their everyday use in the follow-up of patients with diabetes mellitus in telemedicine patient care.

In this study, we apply radiomic feature extraction to fundus photographs in patients with diabetic mellitus, with and without DR in different stages, to identify texture-based biomarkers associated with DR. We first identify novel radiomic-derived fundus photographs that distinguish eyes with no DR from those with non-proliferative DR (NPDR). Additionally, we aim to evaluate how image features vary across different stages of DR and how they change over time as the disease progresses. These results may facilitate follow-up of patients in remote or underserved areas who lack access to OCT and wide-field fundus photography for DR screening.

## Methods

### Data Acquisition

We conducted a retrospective study that used 45-degree fundus photographs to collect high-quality images from patients diagnosed with diabetic mellitus with and without DR. Patients were recruited by the Guerrilla Eye Service, founded in 2005 by the senior author (E.W.) at the University of Pittsburgh School of Medicine. The study adhered to the principles of the Declaration of Helsinki and was approved by the Institutional Review Board of the University of Pittsburgh. The images were initially graded by an ophthalmologist at the time of examination. During study enrollment, a second ophthalmologist independently re-evaluated the images, and any cases with discrepancies in DR staging were excluded from the study.

**Figure 1.**
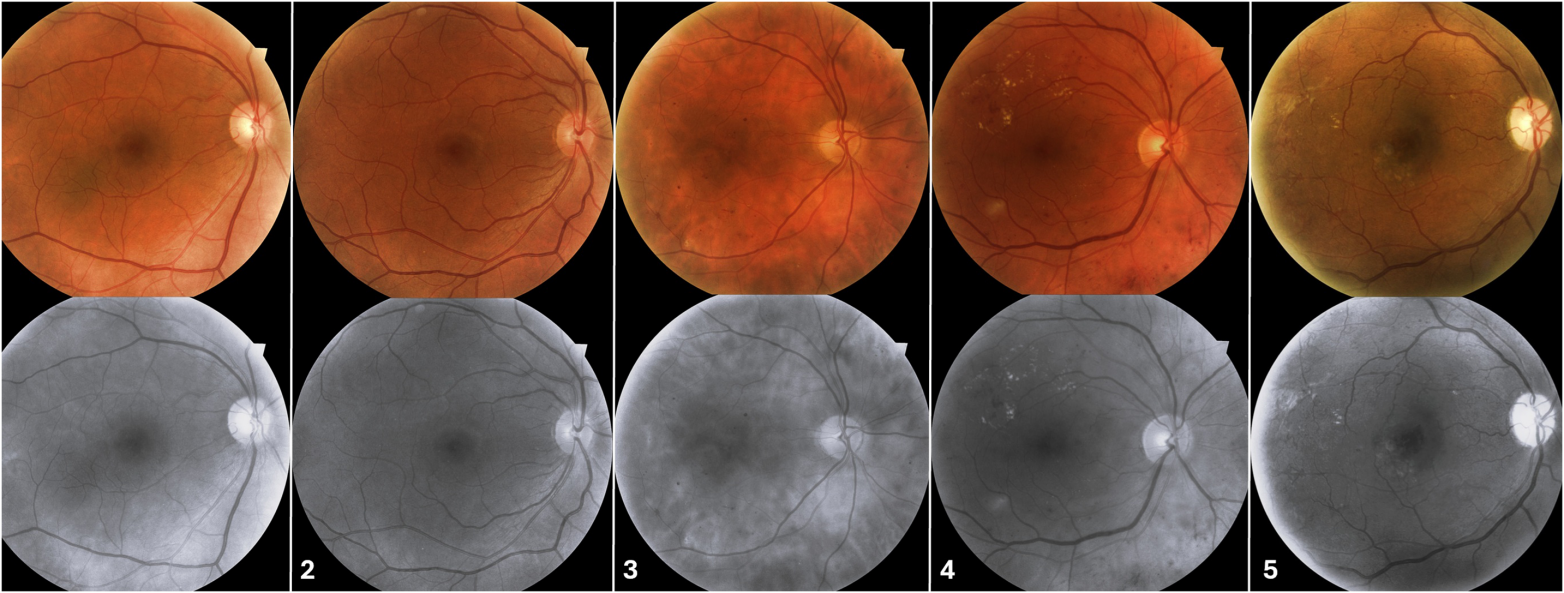
Fundus photography of the right eyes of five patients with diabetes with no diabetic retinopathy (1), mild non-proliferative diabetic retinopathy (NPDR) (2), moderate NPDR (3), severe NPDR (4), and proliferative diabetic retinopathy (PDR) (5). The gray-scale images were used to extract radiomics features.

**Figure 2.**
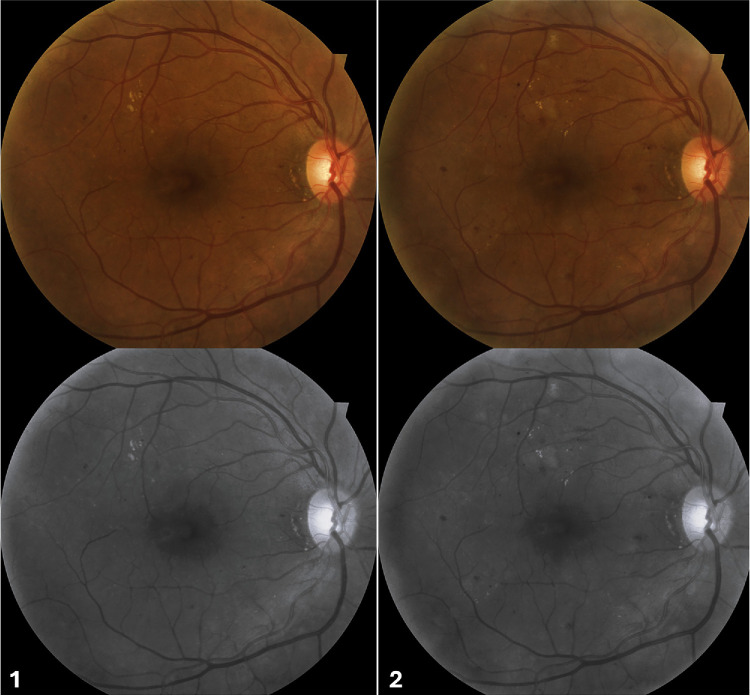
Fundus photography of the right eye with moderate non-proliferative diabetic retinopathy, at baseline (1) and 1 year later (2). The gray-scale images were used to extract radiomics features.

We included 172 fundus photographs by quality assessment, from patients with diabetes mellitus: 35 with no DR, 33 with mild NPDR, 35 with moderate NPDR, 35 with severe NPDR, and 34 with PDR (Fig. 1). Additionally, we analyzed 34 eyes with NPDR that had 2 fundus images obtained over a 1-year follow-up period (Fig. 2). Each participant received a comprehensive ophthalmic examination, including detailed medical history, visual acuity measurement, intraocular pressure assessment, slit-lamp biomicroscopy, and dilated fundus examination to confirm the presence or absence of pathology.

Exclusion criteria were applied to eliminate eyes with other vitreoretinal pathologies, including AMD, uveitis, retinal vascular occlusion, high myopia, epiretinal membrane, or any condition that could affect the fundus images. Images with artifacts or shadows that could interfere with image acquisition or texture analysis were also excluded. All the fundus images had the same dimensions, field of view, and were of consistently high quality.

### Feature Extractions

Radiomic feature extraction was performed on the whole 45-degree fundus images from CenterVue DRSplus (CenterVue S.p.A., Padua, Italy), and each image was captured at a resolution of approximately 10 megapixels (approximately 77–80 pixels per degree of retina), sufficient for detailed analysis of the posterior pole using the Pyfeats python-based radiomics library.^20^ All color fundus images were converted to grayscale prior to analysis by using black-and-white filter using the Windows Photos app on Windows 11. In these images, brighter areas represent highly reflective structures, such as the optic disc and blood vessels, whereas darker regions correspond to less reflective retinal areas. This processing standardized the images for analysis (see Fig. 1). A total of 52 texture features were extracted from each image, encompassing 4 categories: first-order statistics (FOS), gray-level co-occurrence matrix (GLCM), Gabor texture (GT), and Law's texture energy (LTE)^21^^–^^23^:

• FOS features (*n* = 16): Quantified the intensity distribution of pixels, including percentiles (10th, 25th, 75th, and 90th), coefficient of variation, energy, entropy, histogram width, kurtosis, mean, median, mode, skewness, and variance. These measures describe the global brightness and variability within the segmented choroid.
• GLCM features (*n* = 14): Calculated from co-occurrence matrices at a fixed pixel offset and included angular second moment, contrast, correlation, sum of squares variance, inverse difference moment, sum average, sum variance, sum entropy, entropy, difference variance, difference entropy, information measures of correlation, and maximal correlation coefficient. These capture spatial dependencies and textural regularity between neighboring pixels.
• GT (Gabor) features (*n* = 16): Obtained by normalizing pixel values to the range −0.5 to 0.5 and applying Gabor filters with 4 orientations (0 degrees, 45 degrees, 90 degrees, and 135 degrees) and 2 spatial frequencies (0.1 and 0.4 cycles/pixel). The mean and standard deviation of the filtered outputs were computed for each orientation–frequency pair, representing multi-scale, multi-directional texture information.
• LTE features (*n* = 6): Derived using Law's texture energy method with a 3 × 3 convolution mask, capturing localized energy patterns and directional textures.

### Statistical Analysis

We first compared eyes without DR (group 1 = 35 images) to eyes with any stage of NPDR (groups 2–4 = 138 images) to better characterize the differences between normal fundus photographs and those with NPDR. All extracted features were then compared across the five groups to assess trends in feature changes with increasing disease severity. Additionally, we analyzed a subset of 34 eyes with NPDR that had 2 fundus images obtained over a 1 year follow up period. Although their disease stage remained unchanged, these eyes exhibited increased hemorrhages and exudates, allowing us to investigate radiomic feature changes associated with subtle progression in fundus appearance.

All numeric extracted features were compared between early and advanced DR using two-sided Wilcoxon rank-sum tests, with Bonferroni correction applied for multiple comparisons. Features were assigned to one of four predefined families (FOS, GLCM, Gabor, and LTE), and the two features with the lowest adjusted *P* values in each family were selected for visualization. Selected features were standardized using z-scores, and 95% confidence intervals for the median were estimated using 1000 bootstrap resamples. Boxplots with overlaid bootstrapped confidence intervals were generated for the selected features.

To evaluate the discriminatory ability of radiomics features, least absolute shrinkage and selection operator (LASSO)-regularized logistic regression was used to distinguish eyes in group 1 from those in groups 2 to 4. Model performance was assessed using repeated nested stratified cross-validation (5 outer folds and 5 inner folds, repeated 50 times). The regularization parameter was selected in the inner loop based on the area under the receiver operating characteristic curve (AUC) using the λ_₁se_ selection rule. Discrimination was quantified using mean AUC derived from out-of-fold predictions, with empirical 95% uncertainty intervals estimated across repetitions. A Youden threshold was identified from out-of-fold predictions within each repetition, and sensitivity and specificity were averaged across repetitions. Linear regression analysis and multi-class logistic regression were done for the prediction of DR stage. All analyses were performed in R software (version 4.5.1) using the pROC and glmnet packages, as well as Python software.

## Results

Overall, 172 fundus photographs from patients with diabetes mellitus were analyzed, including 35 with no DR, 33 with mild NPDR, 35 with moderate NPDR, 35 with severe NPDR, and 34 with PDR. In addition, 34 eyes with NPDR that had 2 fundus images obtained over a 1 year follow-up period were analyzed. Because the radiomic extraction is fully automated, running the pipeline multiple times on the same image yields identical features, ensuring complete model stability and reproducibility.

### No DR Versus NPDR

**Figure 3.**
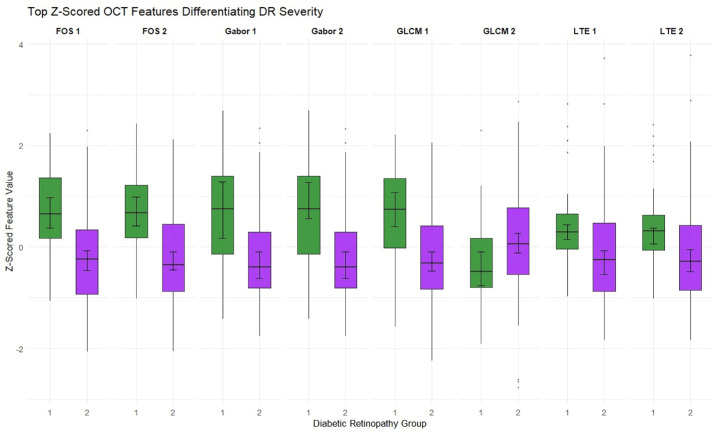
The difference between selected radiomics features from fundus photographs with no diabetic retinopathy (35 images) with green color versus eyes with non-proliferative diabetic retinopathy (138 images) with purple color.

To investigate whether radiomic features could distinguish images without DR from those with NPDR, we compared 35 fundus photographs without DR to 138 photographs with any stage of NPDR (Fig. 3).

• FOS: Of the 16 extracted features, 6 were significantly different between the 2 groups, including FOS_mean (*P* = 0.001), FOS_median (*P* < 0.001), FOS_mode (*P* = 0.007), FOS_coefficient_of_variation (*P* = 0.002), FOS_25 percentile (*P* < 0.001), and FOS_75 percentile (*P* = 0.007).
• GLCM: Among the 14 extracted features, GLCM_sum_average_mean showed a significant difference (*P* = 0.001).
• GT (Gabor) features: Of the 16 features, 13 demonstrated significant differences between the groups, including:
  • GT_th_0_0_freq_0_1_mean (*P* = 0.001), GT_th_0_0_freq_0_4_mean (*P* < 0.001), GT_th_0_0_freq_0_4_std (*P* = 0.005),
  • GT_th_1_0_freq_0_1_mean (*P* = 0.001), GT_th_1_0_freq_0_4_mean (*P* < 0.001), GT_th_1_0_freq_0_4_std (*P* = 0.002),
  • GT_th_2_0_freq_0_1_mean (*P* < 0.001), GT_th_2_0_freq_0_1_std (*P* = 0.034), GT_th_2_0_freq_0_4_mean (*P* < 0.001), GT_th_2_0_freq_0_4_std (*P* = 0.015),
  • GT_th_3_0_freq_0_1_mean (*P* = 0.001), GT_th_3_0_freq_0_4_mean (*P* < 0.001), and GT_th_3_0_freq_0_4_std (*P* = 0.002).
• LTE: Of the 6 extracted features, LTE_ss_3 was significantly different between groups (*P* = 0.047).

The LASSO-regularized logistic regression model used to discriminate eyes in group 1 from those in groups 2 to 4 achieved a mean AUC of 0.742 (95% empirical interval = 0.701–0.775), with a sensitivity of 0.692 (95% empirical interval = 0.592–0.775) and a specificity of 0.755 (95% empirical interval = 0.664–0.879).

### Different Stages of DR

**Figure 4.**
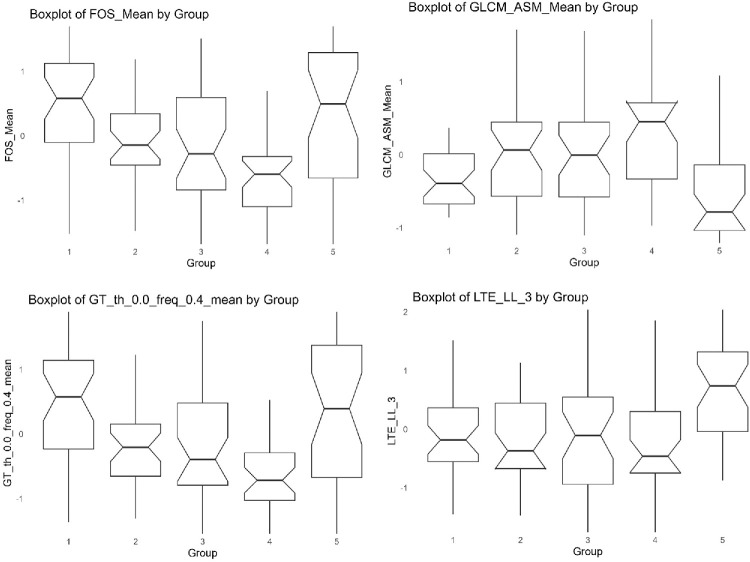
Differences in selected radiomics features across fundus photographs from eyes with no diabetic retinopathy (DR; group 1, *n* = 35), mild NPDR (group 2, *n* = 33), moderate NPDR (group 3, *n* = 35), severe NPDR (group 4, *n* = 35), and proliferative DR (PDR) (group 5, *n* = 34).

To evaluate whether radiomic features could differentiate between stages of DR, we analyzed 35 fundus photographs without DR (group 1), 33 with mild NPDR (group 2), 35 with moderate NPDR (group 3), 35 with severe NPDR (group 4), and 34 with PDR (group 5). A linear trend of feature changes was observed with increasing disease severity from no DR to severe NPDR, whereas PDR exhibited a distinct pattern across all images (Fig. 4).

• FOS: Of the 16 extracted features, 14 were significantly different among the 5 groups, including FOS_mean (*P* = 0.001), FOS_variance (*P* = 0.021), FOS_median (*P* < 0.001), FOS_mode (*P* = 0.010), FOS_skewness (*P* < 0.001), FOS_kurtosis (*P* = 0.007), FOS_energy (*P* < 0.001), FOS_entropy (*P* < 0.001), FOS_coefficient of variation (*P* = 0.006), FOS_10 percentile (*P* < 0.001), FOS_25 percentile (*P* < 0.001), FOS_75 percentile (*P* < 0.001), FOS_90 percentile (*P* = 0.016), and FOS_histogram width (*P* = 0.021).
• GLCM: Among the 14 extracted features, 7 features were statistically significant among all groups including GLCM_ASM_mean (*P* = 0.005), GLCM_sum of squares variance_mean (*P* = 0.024), GLCM_sum average_mean (*P* = 0.001), GLCM_sum variance_mean (*P* = 0.023), GLCM_sum entropy_mean (*P* < 0.001), GLCM_entropy_mean (*P* = 0.001), and GLCM_information 2_mean (*P* = 0.011).
• GT (Gabor) features: Of the 16 features, 11 demonstrated significant differences among the groups, including:
  • GT_th_0.0_freq_0.1_mean (*P* = 0.002), GT_th_0.0_freq_0.4_mean (*P* = 0.001), GT_th_0.0_freq_0.4_std (*P* = 0.012),
  • GT_th_1.0_freq_0.1_mean (*P* = 0.002), GT_th_1.0_freq_0.4_mean (*P* = 0.001), GT_th_1.0_freq_0.4_std (*P* = 0.009),
  • GT_th_2.0_freq_0.1_mean (*P* = 0.003), GT_th_2.0_freq_0.4_mean (*P* = 0.002),
  • GT_th_3.0_freq_0.1_mean (*P* = 0.002), GT_th_3.0_freq_0.4_mean (*P* = 0.001), GT_th_3.0_freq_0.4_std (*P* = 0.007).
• LTE: Of the six extracted features, LTE_LL_3 was significantly different among the five DR stages (*P* = 0.022).

### Linear Regression Analysis for the Prediction of DR Stage From Individual Features in no DR and all NPDR

In this analysis, all DR stages were put as the X variable and each individual feature as the Y variable, and we determined how many were significant for each feature group. The R-squared values are small, but in the first analysis above, we show that combined, they allow for significant classification (Table 1).

**Table 1.** Linear Regression Analysis for the Prediction of Diabetic Retinopathy (DR) Stage From Individual Features in no DR and all Non-Proliferative DR

Features (Linear Regression *R*^2^ Results)	Mean	CI, Low	CI, High	Number of Significant *P* values
FOS	0.088	0.051	0.124	11 out of 16
LTE	0.047	0.022	0.069	4 out of 6
GLCM	0.035	0.017	0.060	6 out of 14
GT	0.105	0.079	0.129	13 out of 16

FOS, first-order statistics; GLCM, gray-level co-occurrence matrix; GT, Gabor texture; LTE, Law's texture energy; CI, confidence interval.

### Multi-Class Logistics Regression to Predict Diabetes Stages

The results show classification accuracy that has non-overlapping confidence intervals with the shuffle control for classification using all features and classification using FOS features alone. The others (LTE, GC, and GLCM) had overlapping confidence intervals, suggesting that they are not significantly better than chance (Table 2).

**Table 2.** Multi-Class Logistics Regression to Predict Diabetes Stages

	Mean	CI, Low	CI, High
All features	0.366	0.322	0.417
All features shuffle	0.214	0.162	0.258
FOS features	0.395	0.346	0.444
LTE features	0.326	0.238	0.403
GT features	0.268	0.218	0.317
GLCM features	0.326	0.244	0.383

### Linear Regression Results for the Prediction of DR Stage From Individual Features in all DR Stages

All DR stages were put as the X variable and each individual feature as the Y variable, and we determined how many were significant for each feature group. The R-squared values are small, but in the first analysis above, we show that combined, they allow for significant classification (Table 3).

**Table 3.** Linear Regression Results for the Prediction of Diabetic Retinopathy (DR) Stage From Individual Features in all DR Stages

Features (Linear Regression *R*^2^ Results)	Mean	CI, Low	CI, High	Number of Significant *P* Values
FOS	0.024	0.013	0.038	6 out of 16
LTE	0.024	0.010	0.040	4 out of 6
GLCM	0.026	0.013	0.038	6 out of 14
GT	0.007	0.004	0.012	1 out of 16

### Longitudinal Analysis

**Figure 5.**
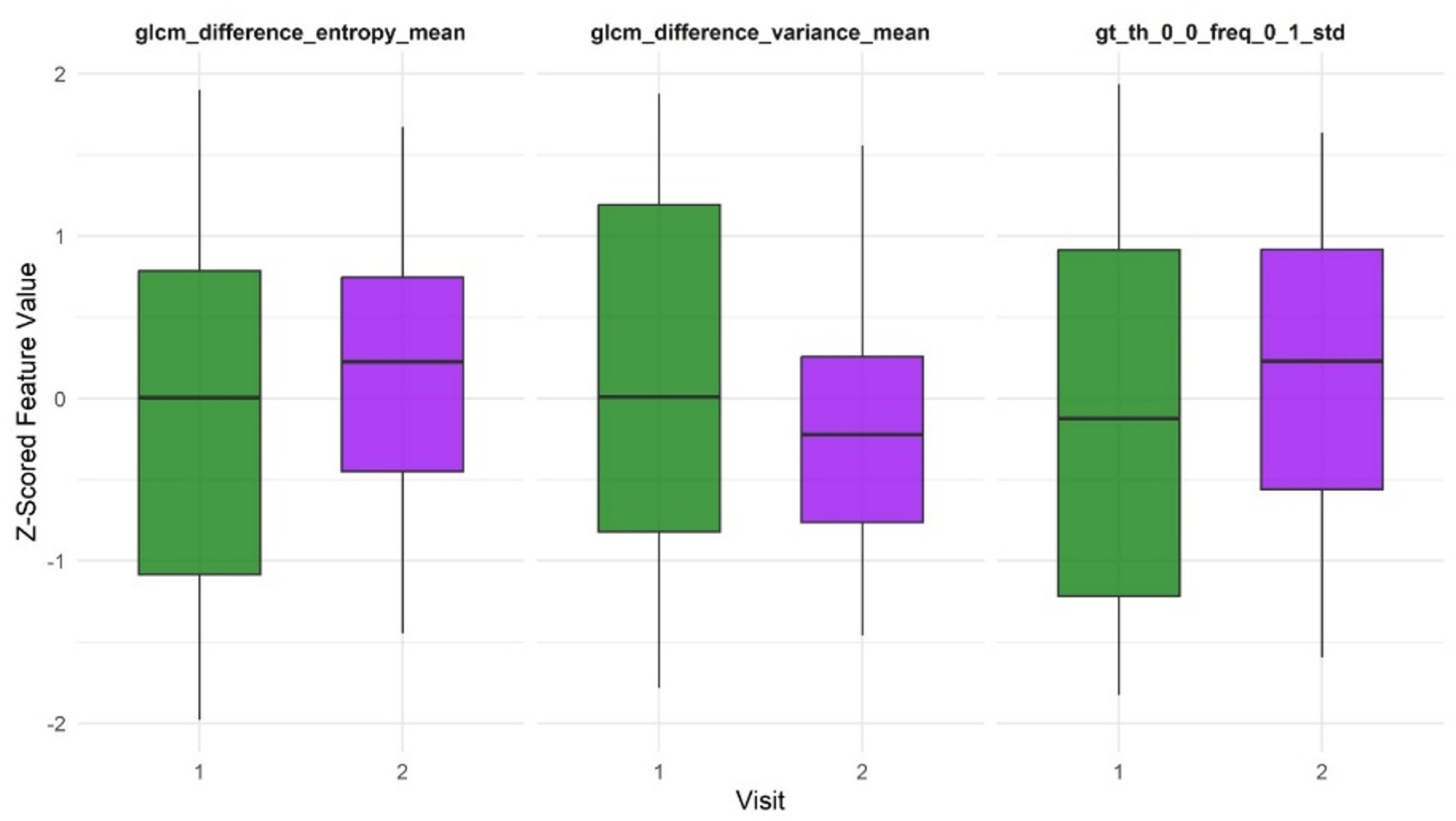
In the follow-up analysis of 34 eyes with NPDR, 3 radiomic features demonstrated significant differences between baseline (*green*) and 1 year of follow-up (*purple*); however, these differences did not remain significant after Bonferroni correction.

To evaluate whether radiomic features could capture subtle progression over time, we analyzed 34 eyes with NPDR that had fundus photographs obtained at baseline and after 1 year of follow-up. Among the 52 extracted features, 3 showed statistically significant differences between the 2 time points: GLCM_difference variance_mean (*P* = 0.020), GLCM_difference entropy_mean (*P* = 0.044), and GT_th_0.0_freq_0.1_std (*P* = 0.033). However, none of these associations remained significant after applying Bonferroni correction (Fig. 5).

The linear regression analysis for the prediction of DR differences from individual features in longitudinal data showed no significant difference between the early and late stages (Table 4).

**Table 4.** Linear Regression Analysis for the Prediction of Diabetic Retinopathy (DR) Differences From Individual Features in Longitudinal Data

Features Visit 1 vs. Visit 2 (Linear Regression *R*^2^ Results)	Mean	CI, Low	CI, High
Train	0.776	0.733	0.818
Test	0.528	0.453	0.608
First visit	2.705	2.147	3.206
Second visit	2.470	1.852	3.029
Second first visit	−0.235	−0.794	0.294

## Discussion

In this study, we extracted and compared radiomic features from fundus photographs of patients with DR. Our analysis showed that radiomics could distinguish fundus photographs with no DR from those with NPDR. Significant differences were also observed across five stages of DR, including no DR, mild, moderate, and severe NPDR, and PDR. However, radiomics was not sensitive enough to detect subtle changes in fundus photographs in eyes with NPDR in a 1-year follow-up. PDR exhibited features that were distinct from the other stages. In this study, radiomics was used as a handcrafted feature extraction framework, in which predefined quantitative descriptors were computed from fundus images. These features were subsequently used as inputs to conventional machine learning classifiers for feature selection and classification. Importantly, no end-to-end feature learning or deep learning-based representation learning was performed, allowing a clear separation between feature engineering and model training.

The rising number of DR cases poses a public health challenge, making regular screening essential. Whereas manual diagnosis is time-consuming, expert-dependent, and subject to interobserver variability, artificial intelligence provides a reliable tool to support clinicians and enhance diagnostic consistency; however, current FDA-approved algorithms remain limited by their time requirements, licensing costs, and narrow disease scope.^8^^,^^9^^,^^19^ Radiomics uses mathematical formulas to analyze grayscale histograms, region-of-interest shapes, and texture-defining matrices.^24^ It offers several advantages over traditional deep learning for feature extraction, including the ability to operate with small datasets, limited computing power, and standardized, interpretable features that allow in-depth study and improved model transparency.^16^ Additionally, its predefined and potentially nonlinear feature transformations are computationally efficient yet flexible. They can be combined with other machine learning classifiers to create interpretable, sample-efficient models applicable across diverse settings.^16^ The identification of DR stages through radiomics has the potential to significantly improve diagnostic accuracy and streamline disease staging, particularly within telemedicine with extensive patient data. Traditional evaluation of fundus photographs by expert ophthalmologists provides valuable insights for screening but is limited by its time-consuming nature and reliance on manual interpretation. In contrast, radiomics enables quantitative analysis of image heterogeneity with minimal assumptions about structure, offering a faster and more detailed assessment of the underlying architecture. Our findings suggest that radiomic features can capture fundus changes associated with DR, indicating potential utility in supporting telemedicine-based patient care.

Radiomics, although initially established in oncology and showing considerable promise, remains relatively underexplored in ophthalmology due to the complexity of ocular imaging and the diversity of disease-specific modalities.^19^^,^^25^ Radiomics has been applied to retinal diseases primarily for diagnostic screening, treatment response prediction, differential diagnosis, staging, and longitudinal monitoring, with particular emphasis on AMD, DR, CSCR, and other macular disorders. Studies to date have leveraged diverse imaging modalities, including OCT, OCTA, fundus photography, and ultra-widefield fluorescein angiography, to extract quantitative features that enhance disease characterization.^13^^,^^16^^,^^17^^,^^19^

Several studies have demonstrated the potential of radiomics in DR detection. Baffa et al. applied a radiomics-based approach to fundus images of patients with mild DR and controls, using a deep neural network classifier, and achieved 94% accuracy and 93.34% sensitivity, highlighting the promise of computer vision in ophthalmology.^26^ Carrera-Escalé et al. evaluated multiple classifiers using OCT, OCTA, and fundus photography for diagnosing DM, DR, and referable DR, finding that OCTA-based models performed best for DR and referable DR, whereas OCT with logistic regression performed best for DM.^13^ Shamsan et al. developed 3 approaches, each combining radiomic features from Dense-121 or Alex models with handcrafted features, achieving early DR detection with 97.92% sensitivity, 99.1% accuracy, 99.4% specificity, and 99.06% precision.^27^ Additionally, Soren et al. applied radiomics analysis to ultra-wide OCTA and observed significant differences in radiomic features across increasing DR severity, from no DR to PDR.^28^ Although most published studies focus on early detection of DR from healthy eyes using wide-field imaging, OCT, or OCTA, in real-world telemedicine practice, such modalities are not universally available, and patient follow-up is most commonly performed using standard fundus photography. Our results demonstrate that radiomic feature extraction from fundus photographs can distinguish patients with no DR from those with NPDR, which may help identify patients with diabetes in telemedicine follow-up who require closer monitoring and timely intervention. We also observed progressive changes in multiple features in different stages of DR from no DR to severe NPDR, although subtle changes during follow-up may not be detectable with this approach. The distinct features observed in PDR compared with other stages may be attributable to massive retinal hemorrhages, laser scars, or tractional retinal detachment, all of which can affect radiomic features.

The main limitations of this study include its retrospective design, relatively small sample size, single device, single-region setting, and variability in image quality due to intraocular lens status, media opacity, or subtle cataract changes, which may alter fundus image texture, lack of wide-field imaging, and short follow-up period, during which subtle fundus changes occurred, but no DR stage progression was observed. Nevertheless, as a proof-of-concept demonstration of radiomics for identifying pathology in fundus photographs, several measures, such as using standardized radiomics features and a simple Wilcoxon rank-sum test, were implemented to reduce model complexity and overfitting; however, larger and longer-term studies, comparing the performance of additional machine learning models to provide a more comprehensive assessment of radiomic-based classification will be needed to validate and generalize these findings.

## Conclusions

Overall, this study demonstrates that radiomic texture analysis can identify features distinguishing diabetic patients with and without NPDR in fundus images and reveal significant differences across DR stages, from no DR to mild, moderate, severe NPDR, and PDR; however, it was not sensitive enough to detect subtle changes during short-term follow-up. These findings suggest that radiomic features can capture fundus alterations associated with DR, with possible relevance for telemedicine applications.
